# Assessing the Microbial Quality of Shrimp (*Xiphonaeus kroyeri)* and Mussels (*Perna perna*) Illegally Sold in the Vitória Region, Brazil, and Investigating the Antimicrobial Resistance of *Escherichia coli* Isolates

**DOI:** 10.3390/antibiotics13030242

**Published:** 2024-03-06

**Authors:** Daniella Tosta Link, Gustavo Guimarães Fernandes Viana, Lívia Pasolini Siqueira, Carolina Magri Ferraz, Romário Alves Rodrigues, Luis Antonio Mathias, Marita Vedovelli Cardozo, Gabriel Augusto Marques Rossi

**Affiliations:** 1 Department of Veterinary Medicine, University of Vila Velha (UVV), Vila Velha 29102-920, ES, Brazil; daniellalink@outlook.com (D.T.L.); gustavo.zero98@gmail.com (G.G.F.V.); siqueiralivia@outlook.com.br (L.P.S.); carolina.ferraz@uvv.br (C.M.F.); 2 Department of Pathology, Reproduction and One Health, São Paulo State University (UNESP), Jaboticabal 14884-900, SP, Brazil; romario.a.rodrigues@unesp.br (R.A.R.); la.mathias@unesp.br (L.A.M.); 3 Microorganism Physiology Laboratory, Department of Biomedical Science and Health, Universidade do Estado de Minas Gerais (UEMG), Passos 37900-004, MG, Brazil; marita.cardozo@uemg.br

**Keywords:** β-lactams, colistin, disk diffusion, enterobacteria, ESBL, mesophiles, psychrotrophs, seafood, shellfish, tetracycline

## Abstract

The consumption of seafood is crucial for food security, but poor hygiene along the food production chain can result in low microbiological quality, posing significant risks for public health and seafood quality. Thus, this study aimed to assess the microbiological quality and antimicrobial sensitivity of *E. coli* from 69 samples of illegally marketed shrimp and mussels in the Vitória Region, Brazil. These foods exhibited poor microbiological quality due to high counts of mesophilic, psychrotrophic, and enterobacteria microorganisms. While this issue is widespread in this area, shrimp samples displayed higher microbial counts compared to mussels, and fresh mussels had elevated counts of enterobacteria compared to frozen ones. Among the 10 *E. coli* isolates, none carried the genes *blaCTX-M-1*, *blaCTX-M-2*, *blaCTX-M-3*, *blaCTX-M-15*, *mcr-1*, *mcr-2*, *mcr-3*, *mcr-4*, and *tet*, associated with antibiotic resistance. Phenotypical resistance to tetracycline and fosfomycin was not observed in any isolate, while only 20% demonstrated resistance to ciprofloxacin. Regarding ampicillin and amoxicillin with clavulanic acid, 60% of isolates were resistant, 10% showed intermediate susceptibility, and 30% were sensitive. One isolate was considered simultaneously resistant to β-lactams and quinolones, and none were conserved as ESBL producers. These findings highlight the inherent risks to local public health that arise from consuming improperly prepared seafood in this area.

## 1. Introduction

The consumption of seafood is of paramount importance for the food security of various populations worldwide, and these foods can originate from either fishing or aquaculture. Among the aquatic animals consumed, in addition to fishes, the consumption of shrimp and mussels stands out, with the production and commercialization of these animals being significant economic activities in some regions of the world [1], such as in the Vitória Region, Brazil. Due to the risk of these animals being able to transmit diseases when contaminated by pathogenic microorganisms and the risk of rapid spoilage due to their intrinsic characteristics [2], in Brazil, since 1950, it has been mandatory for them to undergo sanitary inspection for their commercialization [3]. However, this is often not observed, and these animals are frequently caught and sold directly to the consumers without any proper sanitary inspection in street market establishments. Typically, in these establishments, poor hygienic handling practices are observed, as has been reported in the Vitória Region, which can further compromise the quality and safety of the marketed food [4]. When shrimps and mussels are mishandled during their capture/rearing, processing, or commercialization, they can become contaminated, leading to two distinct sets of consequences: spoilage due to high counts of deteriorating microorganisms and risks to public health due to the transmission of several pathogens, such as the bacterium *Escherichia coli*, among others [5,6].

*Escherichia coli* is a bacterium that assumes critical significance when transmitted through the consumption of contaminated food, underscoring the importance of food safety measures. When present in contaminated food, *E. coli* can lead to severe health implications. Common clinical signs of *E. coli* infection resulting from food consumption include abdominal cramps, diarrhea, and nausea. In some cases, more serious complications, such as hemolytic uremic syndrome (HUS), can occur, posing a significant threat to human health [7]. Therefore, understanding and implementing rigorous hygiene practices in the commercialization of seafood is imperative to mitigate the risk of *E. coli* transmission and safeguard public health.

Additionally, the emergence of antibiotic-resistant strains of *E. coli* poses a significant threat to public health, underscoring the critical importance of understanding and addressing antibiotic resistance. When *E. coli* strains become resistant to commonly used antibiotics, the effectiveness of standard medical treatments is compromised, leading to increased difficulty in managing infections. This resistance can be transmitted to humans through the consumption of food contaminated with antibiotic-resistant isolates, thereby amplifying the risk to public health. The potential consequences include prolonged illness, limited treatment options, and an elevated risk of severe complications [8]. In 2019, an estimated 4.95 million deaths were linked to bacterial antimicrobial resistance (AMR), with 1.27 million deaths specifically attributed to bacterial AMR. *Escherichia coli* emerged as the primary pathogen responsible for fatalities associated with resistance, marking a concerning trend in the impact of antimicrobial resistance on global health [9]. Mitigating the spread of antibiotic-resistant *E. coli* necessitates a multifaceted approach including the prudent use of antibiotics in both medical and livestock/agriculture settings, enhanced surveillance, and robust food safety measures to minimize the transmission of resistant strains through the food chain [10].

There are no studies evaluating the microbiological quality of mussels and shrimp marketed to the population in the Vitória Region, Brazil, nor are there any data on the antimicrobial resistance profile of the *E. coli* derived from these foods. Thus, this research aimed to assess the microbiological quality of illegally marketed shrimp and mussels in the Vitória Region, Brazil. Additionally, the antimicrobial resistance profile of *E. coli* isolates was assessed using the disk diffusion method and by verifying the presence of some genes related to resistance to extended-spectrum beta-lactamases, colistin, and tetracycline. Therefore, this study provides data for various stakeholders that can be used in decision-making and risk reduction processes to safeguard public health.

## 2. Results

In the shrimp samples, the maximum and minimum counts of mesophilic, psychrotrophic, and enterobacteria microorganisms were 6.9 × 10^7^ and 3.2 × 10^3^, 1.0 × 10^10^ and 2.5 × 10^5^, and 4.4 × 10^7^ and 3.0 × 10^1^, respectively. For the evaluated mussel samples, the maximum and minimum counts of mesophilic, psychrotrophic microorganisms, and enterobacteria were 1.6 × 10^8^ and 8 × 10^2^, 9.1 × 10^8^ and 1.2 × 10^4^, and 7.7 × 10^7^ and none detected, respectively.

The means were significantly higher, showing statistical differences, according to the Wilcoxon rank sum exact test, regarding the counts of mesophilic microorganisms (*p* = 0.01357), psychrotrophic microorganisms (*p* = 1.146 × 10^−7^), and enterobacteria (*p* = 0.0002042) when comparing all shrimp samples to the mussel samples in this study. Specifically, the mean values for the mesophilic, psychrotrophic, and enterobacteria counts in the shrimp samples were 6.6, 8.5, and 4.7 log, respectively, whereas for the mussel samples, they were 5.8, 6.4, and 2.7 log.

Furthermore, the mean values for the mesophilic, psychrotrophic, and enterobacteria counts in the frozen mussel samples were 5.8, 6.5, and 2.3 log, respectively, whereas in the fresh mussel samples, they were 5.8, 6.3, and 3.9 log. Notably, there was a statistical difference (*p* = 0.0233) only for the mean counts of enterobacteria, which were higher in the fresh samples.

The results regarding the mean counts of mesophilic and psychrotrophic microorganisms and enterobacteria in the evaluated shrimp and mussel samples from different cities can be found in Table 1.

There were no significant differences (*p* > 0.05) among the municipalities of origin of the samples regarding the mean counts of mesophilic and psychrotrophic microorganisms in shrimp samples from Vila Velha, Vitória, and Serra. However, the mean counts of enterobacteria in the shrimp samples from Serra were significantly higher compared to those from Vila Velha (*p* = 0.02484). Additionally, concerning the mussel samples analyzed in this study originating from three municipalities (Vila Velha, Vitória, and Cariacica), no statistically significant differences were detected in relation to the mean counts of any of the three groups of indicator microorganisms evaluated.

Among the 69 samples analyzed in this study, colonies suggestive of *E. coli* were identified in 20 samples on EMB agar. Subsequently, these colonies underwent confirmation through the PCR technique, revealing that 10 (14.5%, 10/69) of them were indeed the *E. coli* bacterium. Among these 10 isolates, 3 originated from shrimp samples, and 7 originated from mussels. Furthermore, these isolates underwent a PCR to detect the presence of genes such as *blaCTX-M-1*, *blaCTX-M-2*, *blaCTX-M-1*, *blaCTX-M-3*, *blaCTX-M-15*, *mcr-1*, *mcr-2*, *mcr-3*, *mcr-4*, and *tet*, none of which were detected in any of the isolates.

The resistance of these 10 isolates to five different antibiotics [amoxicillin + clavulanic acid (30 µg) (AMC), ampicillin (10 µg) (AMP), ciprofloxacin (5 µg) (CIP), fosfomycin (50 µg) (FOS), and tetracycline (30 µg) (TET)] was assessed using the disk diffusion method. None of the isolates exhibited resistance to tetracycline or fosfomycin, and only two (20%) demonstrated resistance to ciprofloxacin, both of which originated from mussel samples sold in Vitória city. In the case of both ampicillin and the combination of amoxicillin with clavulanic acid, six isolates exhibited resistance (60%), one showed intermediate susceptibility (10%), and three were sensitive (30%) (Table 2). Only one isolate (10%) (from Vitória) was considered as resistant to three antibiotics (AMC, AMP, CIP), three isolates (30%) were resistant to two antibiotics, and four isolates (40%) were resistant to one antibiotic. The isolate resistant to three antibiotics was not considered as multi-drug-resistant (MDR) because the resistance was for two groups of antibiotics (β-lactams and quinolones). Only one isolate, from Vila Velha, was considered sensitive to all the antibiotics tested.

## 3. Discussion

The current Brazilian legislation does not establish the maximum limits allowed for the counts of mesophilic, psychrotrophic, and enterobacteria microorganisms in shrimps and mussels sold to the population. Therefore, it is not possible to assess whether the analyzed samples comply with the current national legislation [12]. However, the International Commission on Microbiological Specification for Foods (ICMSF) recommends that in these foods, the mesophilic microorganism counts should be below 6.0–7.0 Log10 [13], with values above this limit possibly indicating inadequate hygienic practices in the production chain or the poor storage of these foods. Thus, the mean counts (Table 1) in the shrimp and mussel samples from this study demonstrate that the sale of these shrimp and mussels with poor microbiological quality was occurring in all municipalities, possibly indicating that these foods were already deteriorating. Similarly, the high counts of psychrotrophic microorganisms and enterobacteria reinforce the results that poor hygiene and conservation practices were present throughout the production/capture, distribution, and commercialization of the seafood sampled (Table 1). There were no statistically significant differences detected among the three groups of microorganisms indicators used in this study across the various sampled municipalities, indicating a widespread issue, except for the enterobacteria counts in the samples from Vila Velha and Serra. This observation may be attributed to poor hygiene and conservation practices being consistently applied across the study areas or to the likelihood that shrimp and mussels often originate from the same fishing/capturing areas and are merely sold in different municipalities. In this study, only the point of sale of these seafood products was considered, and no information was collected regarding the product’s origin (fishing or capturing area).

In a study carried out in São Paulo, Brazil [14], researchers examined the microbiological quality of 42 samples of chilled shrimp sold in street stalls. They found that the average counts of mesophilic and psychrotrophic microorganisms were 5.4 × 10^7^ and 1.1 × 10^11^, respectively. Additionally, in other regions of Brazil, studies have detected mussel samples with poor microbiological quality and the presence of potentially pathogenic agents [15,16]. This underscores the need for actions aimed at improving the quality of both seafood products, mussels and shrimp, in the commercial seafood market in Brazil, with shrimp samples being significantly more contaminated. Seafood can be contaminated by various sources of contamination throughout the entire production chain, beginning in the environment where these foods are caught or harvested, though contamination can occur during their handling and commercialization [4,17,18]. Therefore, to improve the microbiological quality of the seafood sold in this area, measures focusing on the quality of the water where these animals are caught, with an emphasis on reducing pollution, could be adopted [19]. Additionally, implementing Good Manufacturing Practices (GMPs) during handling and commercialization activities carried out by street stall vendors is essential. This includes better sanitation of equipment and surfaces, appropriate personal hygiene standards, improved temperature control in relation to the products, practices to prevent the presence of animals and insects, and other GMPs [4]. The local health authorities in these municipalities could carry out health education interventions for street stall vendors to improve their awareness of the adoption of GMPs and, consequently, the microbiological quality of these seafood products. Additionally, besides preventive health education actions, a higher frequency of fiscalization is necessary to prevent deteriorating or high-risk foods from being accessible for purchase by the populations of these municipalities.

The counts of mesophilic, psychrotrophic, and enterobacteria microorganisms were significantly higher in samples originating from shrimp compared to those originating from mussels. These results were surprising, as we expected the opposite outcome due to mussels being filter feeders capable of retaining various impurities, including microorganisms, from the aquatic environment [19]. However, some practices during the handling and commercialization of these products help explain the results. Firstly, mussels, after being captured, are handled by fishermen who place them in boiling water for approximately 10 min to open their shells, extract the animals’ meat, and often pack them directly into plastic bags for sale to consumers, keeping them refrigerated or in boxes with ice. This thermal treatment may contribute to reducing the populations of microorganisms present in the food. On the other hand, captured shrimp undergo no thermal treatment and are sometimes manipulated in some locations to remove their shells. Once shell-less, they are usually sold loose on ice or frozen, making them more exposed to sources of contamination.

A possible explanation for the higher rate of contamination of enterobacteria in the fresh mussel samples (3.9 Log) compared to the frozen ones (2.3 Log) may stem from the time elapsed between harvest and consumption/selling, alongside the storage conditions. During the period in which fresh mussels are stored at room temperature or refrigerated, there is an extended window for microbial growth and subsequent bacterial contamination. Fresh mussels are often subject to higher temperatures than their frozen counterparts, fostering an environment conducive to bacterial proliferation. Even under refrigeration, temperatures might not be low enough to entirely hinder bacterial growth, particularly if mishandled. In contrast, the freezing process can aid in maintaining the bacterial loads [20]. However, the counts of mesophilic microorganisms in both groups were the same. Moreover, fresh mussels are susceptible to handling by multiple individuals throughout the supply chain, spanning from harvest to the point of sale, thereby augmenting the likelihood of cross-contamination by spoilage and pathogenic bacteria. This helps to explain these results.

Regarding antimicrobial resistance, succinctly, no resistance to fosfomycin (disk-diffusion), colistin (*mcr-1*, *mcr-2*, *mcr-3*, *mcr-4*), and tetracycline (disk-diffusion and *tet*) was observed. Only one isolate was sensitive to the five antibiotics used in the disk-diffusion test. However, six isolates were resistant to β-lactams (AMC and AMP), and two to quinolones (CIP), as ascertained by the disk diffusion test, but none of the isolates carried the studied genes related to ESBL production (*blaCTX-M-1*, *blaCTX-M-2*, *blaCTX-M-1*, *blaCTX-M-3*, and *blaCTX-M-15*). Resistance to tetracycline is a global concern, despite the widespread use of this antibiotic in human and animal healthcare throughout Brazil. Nevertheless, tetracycline is not registered for aquatic animal use in the country. Although *E. coli* strains derived from shrimp and mussels might not possess inherent tetracycline resistance, they could acquire it through mechanisms like horizontal gene transfer and exposure to environmental antibiotics, followed by the natural selection of resistant strains [21]. Bivalves can serve as biomonitoring organisms for antibiotic residue presence in aquatic settings [22], potentially fostering tetracycline-resistant isolate occurrence, which was not detected in our analysis. Other studies in Brazil examining the resistance profile of *Vibrio* bacteria from seafood or aquatic environments also failed to identify tetracycline resistance [23,24]. However, studies in Brazil focusing on *E. coli* obtained from urban park surface water [25], ready-to-eat foods [26], and unpasteurized milk cheese [27] revealed tetracycline resistance within this microorganism. Consequently, it can be inferred that the absence of tetracycline use in animals and aquatic environments and the low levels of residues within the evaluated area contribute to the absence of tetracycline in *E. coli.*

The same logic can be applied to the absence of resistance to fosfomycin, to which no isolate was resistant, and colistin due to the non-detection of the *mcr-1*, *mcr-2*, *mcr-3*, and *mcr-4* genes in any isolate. Fosfomycin is a useful antibiotic in the context of increasing antibiotic resistance, and it acts by inhibiting bacterial cell wall synthesis. Acquired resistance involves modifications of membrane transporters, the acquisition of plasmid-encoded genes that inactivate fosfomycin, and mutations [28]. It is used in Brazil for treating bacterial infections in humans and animals. Reports exist in Brazil regarding the presence of fosfomycin-resistant *E. coli* in broiler chickens [29], where the antibiotic is used in animal husbandry, as well as in the commercialized carcasses of these animals [30] and in hospitalized patients [31], demonstrating a relationship between fosfomycin use and the occurrence of resistance in *E. coli*. Regarding colistin, a very important antibiotic for the treatment of human infections, the manufacturing, commercialization, and use of zootechnical performance-enhancing additives for farm animals containing this antibiotic have been prohibited in the country since 2018. Resistance levels for this antibiotic in *E. coli* are often very low, although higher levels have been observed in Asia [32]. Nevertheless, *E. coli* resistant to colistin have been reported in Brazil in samples of kale crops [33], broiler chickens and free-range layer hens [34], and poultry carcasses [35]. The non-use of fosfomycin and colistin in aquatic animals such as mussels and shrimp and their consequent absence in aquatic environments may help explain the results of this study.

However, in this study, two isolates of *E. coli* were resistant to ciprofloxacin (quinolones). Ciprofloxacin functions by inhibiting DNA gyrase and topoisomerase IV, enzymes crucial for bacterial DNA replication and maintenance [36]. The mechanisms underlying resistance encompass two primary categories: genetic mutations and the acquisition of resistance-conferring genes. Mutations can impact drug target enzymes, leading to diminished drug binding to the enzyme–DNA complex. Additionally, mutations may occur in regulatory genes governing the expression of efflux pumps located within bacterial membranes, further contributing to resistance development [37]. The quinolone class is of significant importance in both human and veterinary medicine in Brazil. Widely utilized for treating urinary tract infections, its extensive use contributes to the emergence of resistance within *E. coli* isolates [38,39]. Alongside the use of quinolones in human medicine, reducing their use in food-producing animals could potentially mitigate the dissemination of fluoroquinolone resistance among Gram-negative bacterial species [40]. However, the release of most antibiotics into the environment occurs through various pathways, mainly due to incomplete absorption and excretion by humans and animals. This environmental contamination, particularly in marine ecosystems, can exert selective pressure on bacterial communities, fostering the development of antibiotic-resistant strains or the proliferation of resistance genes [41]. The global presence of quinolone resistance genes in aquatic environments has been extensively documented, especially in areas impacted by urban discharges [42]. In the Vitória Region, there is frequent deposition of untreated effluents directly into the waters where seafood collection often takes place. This set of information helps to elucidate the occurrence of quinolone resistance observed in this study.

Six isolates from this study showed resistance to amoxicillin with clavulanic acid, while six isolates demonstrated resistance to ampicillin, both belonging to the class of β-lactam antibiotics. Additionally, four isolates exhibited simultaneous resistance to both antibiotics. However, none of the isolates were considered ESBL producers due to the absence of the *blaCTX-M-1*, *blaCTX-M-2*, *blaCTX-M-3*, and *blaCTX-M-15* genes. β-lactams are widely employed in the treatment of a variety of bacterial infections in both humans and animals, representing the oldest and most widely used group of antibiotics in medical history. The bacterium *E. coli* has the capability to develop various mechanisms of resistance to β-lactams, such as enzyme production, the overexpression of efflux pumps, and the modification of porins. However, resistance to these compounds is often associated with the presence of β-lactamases, enzymes capable of hydrolyzing the β-lactam ring of antibiotics, rendering them inactive. Some isolates may produce ESBL, which affects a broad range of antibiotics, and are mainly encoded on plasmids. These mobile genetic elements present in bacteria found in aquatic and soil environments can accelerate the transfer of ESBL genes to animals and humans [43]. In this study, the resistance observed to β-lactams may have occurred due to mechanisms other than ESBL production. However, β-lactam antibiotics are commonly reported in aquatic environments mainly due to wastewater, sewage, and farm discharges as common contamination sources [44]. This contributes to the detection of isolates of *E. coli* resistant to β-lactams, which have been widely reported in Brazil, such as in aquatic environments [45,46], sea fishes [47], bivalves [48,49,50], and shrimp [51], highlighting the potential risks to public health, as shown by our data.

Finally, the contamination of bivalves and shrimp by antibiotic-resistant *E. coli* may have occurred due to poor water quality related to environmental pollution in the area, as discussed. However, it is worth noting that the contamination of these foods may also have occurred through the adoption of poor hygiene practices during their distribution and commercialization [52,53]. The lack of Good Manufacturing Practices (GMPs) in this area has been made evident [4]. This also explains the high counts of mesophilic, psychrotrophic, and enterobacteria microorganisms found in this study.

We would like to highlight some limitations of this study: (1) We experienced difficulties in standardizing sample collection due to the environmental regulations in the area, resulting in fluctuations in product commercialization during the study period. (2) Colistin resistance was assessed by PCR without performing antimicrobial susceptibility testing; moreover, the antibiotics AMP, CIP, and FOS were only tested using antimicrobial susceptibility testing without PCR for gene detection. (3) Only four classes of antibiotics were used in this study, which hindered the detection of MDR isolates by the employed criteria. (4) The species of the other ten isolates that appeared as metallic green on EMB agar were not identified and were not confirmed as *E. coli* by PCR; thus, they were excluded from this study, potentially representing other clinically relevant enterobacteria.

## 4. Materials and Methods

### 4.1. Sampling Scheme

**Figure 1:**
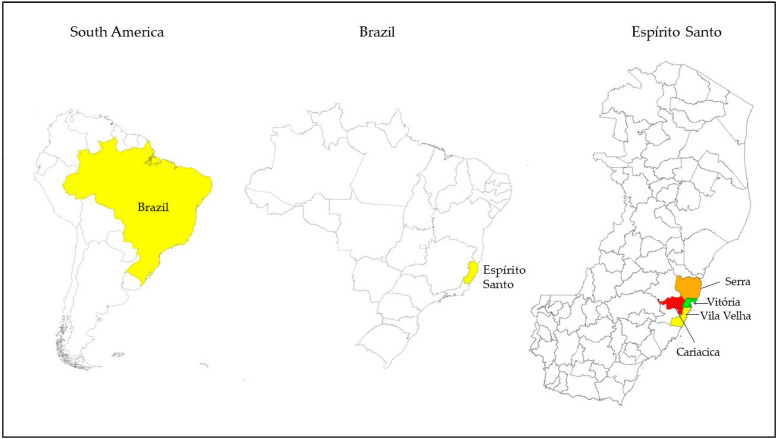
Map of the area where samples of shrimp and mussels were acquired during the year 2023 for microbiological analysis. Samples from the municipalities of Cariacica (mussels), Serra (shrimps), Vila Velha, and Vitória (mussels and shrimp) were included. These municipalities are part of the Vitória Region (the capital city’s macroregion), located in the state of Espírito Santo, Brazil.

The Vitória Region is located in the state of Espírito Santo, Brazil. This region is the most economically developed in the state and has the largest population in the state. In this study, four municipalities belonging to this macroregion were included for sample collection: Cariacica, Serra, Vila Velha, and Vitória (the state capital) (Figure 1).

The samples were acquired from between February and August 2023 through direct purchases from traders predominantly consisting of street stall vendors who sold shrimp or mussels without the presence of a seal of approval from any regulatory agency responsible for the sanitary inspection of these products (illegal). Thus, when already packaged for sale, as was the case with mussels, one package (approximately 500 g to 1 kg) was purchased from the trader. In instances where sales were made in bulk, a frequent occurrence with shrimp, approximately 250 g of the product was acquired.

A set of 69 samples was used in this study. The shrimp (*Xiphonaeus kroyeri*) samples were acquired from 15 street stalls, with 5 located in Serra, 5 in Vila Velha, and 5 in the Vitória Region. Two samples were acquired from each street stall, with the sampling interval being approximately 15 days, totaling 30 samples of frozen shrimp.

Regarding mussels (*Perna perna*), 39 samples were acquired from 20 different establishments. A set of 21 samples were obtained from Vitória, 4 from Cariacica, and 14 from Vila Velha. Five samples were collected from 1 street stall, three samples from 3 different street stalls, two samples from 9 street stalls, and a single sample from 7 different street stalls. Ten samples were fresh mussels, and twenty-nine samples were frozen. In this study, due to the difficulty in collecting samples during certain times of the year owing to fishing/collecting bans due to current environmental legislation samples sold fresh (in the presence of ice) or frozen were included. The lack of standardization in sample acquisition, both in terms of their presentation (fresh or frozen) and the quantity of samples per municipality, occurred due to the large variation in availability during the year.

### 4.2. Microbiological Analyses

After purchasing the samples, they were kept in their original commercial packaging and transported in an insulated box with reusable ice to the laboratory. The packages underwent external disinfection with 70% ethyl alcohol before being opened. Subsequently, they were aseptically opened and homogenized. Next, 25 g of each sample was withdrawn and subjected to decimal serial dilution using sterile 0.1% peptone water to prepare the 10^−1^ dilution and subsequent dilutions.

This study determined the counts of populations of mesophilic microorganisms, psychrotrophic microorganisms, and enterobacteria and isolated colonies suggestive of *E. coli.* The cultivation of mesophilic bacteria was performed using the pour plate technique on Plate Count Agar (PCA) (Kasvi^®^, Curitiba, Brazil) according to APHA 08:2015 [54]. In this method, 1 mL of each dilution was added to a sterile Petri dish, and then 15 mL of liquid agar was poured onto it. Subsequently, these plates were incubated at a temperature of 35 ± 1 °C for 24–48 h. For the analysis of psychrotrophic bacteria counts, 100 µL of each dilution was added to the surfaces of plates containing PCA and distributed using a Drigalski loop. The plates were incubated at 7 ± 1 °C for 7 days, as indicated by the APHA 13.61:2015 method [55].

To perform enterobacteria counting, the APHA 9.62:2015 method [56] was used, wherein 1 mL of each dilution was added to Violet Red Bile Glucose (VRBG) agar (Kasvi^®^, Curitiba, Brazil). The pour plate technique was employed, but with the addition of an agar overlay on the surface of the previously solidified agar. These plates were incubated at a temperature of 35 ± 1 °C for 18–24 h. In the counting, colonies with a red–purple color that were at least 0.5 mm in diameter and surrounded by a reddish halo indicative of bile salt precipitation were counted. Additionally, for the verification of the presence of *E. coli*, 200 µL of the 10^−1^ dilution was inoculated onto the surfaces of plates containing Eosin Methylene Blue (EMB) agar (Kasvi^®^, Curitiba, Brazil) and incubated at a temperature of 35 ± 1 °C for 24 h. Colonies suggestive of *E. coli* were those presenting a shiny metallic green coloration in the culture medium, which were streaked onto another plate of EMB agar for isolation and then transferred to sterile Eppendorf tubes containing 1 mL of Brain Heart Infusion (BHI) broth and 300 μL of glycerol for subsequent cooling over a period of 1 h at 8 °C, followed by freezing at −25 °C. Posteriorly, these colonies were confirmed via Polymerase Chain Reaction (PCR).

### 4.3. Polymerase Chain Reaction (PCR)

#### 4.3.1. DNA Extraction

DNA extraction from bacterial isolates was based on the protocol proposed by Bag and colleagues [57]. For this purpose, 1.0 mL aliquots of bacterial culture from each isolate were centrifuged at 10,000× *g* for 3 min to obtain a cell pellet. The culture medium was discarded, and the bacterial pellet was resuspended in 450 µL of extraction buffer [Tris-HCl 160 mM pH 8.0; EDTA 50 mM pH 8.0; NaCl 20 mM; and SDS 0.5% (*w*/*v*)]. Cell lysis occurred in a Thermomixer dry bath (Eppendorf, Hamburg, Germany) at 65 °C for 30 min. Subsequently, 180 µL of 5 M potassium acetate was added to the solution, which, after homogenization, was kept in ice for 15 min. Purification was performed with 400 µL of chloroform/isoamyl alcohol 24:1 (*v*/*v*) under centrifugation at 12,000× *g* for 10 min. The supernatant was transferred to new tubes, to which 1000 µL of chilled absolute ethanol was added. The solution was mixed and kept in a freezer at −20 °C for 12 h for DNA precipitation. Subsequently, the tubes were centrifuged at 12,000× *g* for 18 min to obtain the DNA pellet, which was washed with 1000 µL of 70% (*v*/*v*) ethanol, dried in an oven, and resuspended in 30 µL of TE buffer 10:1 (Tris-HCl 10 mM pH 8.0; EDTA 1 mM pH 8.0). DNA samples were quantified using a NanoDropOne spectrophotometer (Thermo Scientific, Waltham, MA, USA).

#### 4.3.2. *E. coli* Identification

For the confirmation of the *E. coli* species, the protocol described by Tsen and colleagues [58] was utilized with the following adaptations: 95 °C for 3 min, followed by 35 cycles (94 °C for 20 s, 56 °C for 30 s, 72 °C for 30 s), and a final extension step at 72 °C for 2 min.

#### 4.3.3. Detecting Genes Related to Antimicrobial Resistance

A PCR was also employed to verify the presence of genes related to antibiotic resistance. The presence of the *blaCTX-M-1*, *blaCTX-M-2* [59], *blaCTX-M-1*, *blaCTX-M-3*, and *blaCTX-M-15* [60], associated with extended-spectrum β-lactamase (ESBL) production; *mcr-1*, *mcr-2*, *mcr-3*, and *mcr-4* [61], related to colistin resistance; and *tet* [62], associated with tetracycline resistance, was confirmed.

### 4.4. Phenotypical Resistance/Sensibility of E. coli Isolates

The confirmed *E. coli* isolates, which were stored frozen, were reactivated for the evaluation of their susceptibility/resistance to antimicrobials using the disk diffusion method [63]. For this purpose, they were inoculated on Nutrient Agar (Kasvi, Brazil) for reactivation and incubated at 37 °C for 24 h to prepare the inocula.

The inocula were prepared in tubes containing 3 mL of 0.85% saline solution, with the solution being turbidified with the culture until an OD_625_ nm of 0.1 to 0.2 nm was reached. Subsequently, these inocula were streaked onto plates containing Mueller–Hinton agar (Kasvi, Curitiba, Brazil) using a sterile swab, and disks containing selected antimicrobial agents were placed on the plates. The antimicrobial groups used were as follows: (1) β-lactams − amoxicillin + clavulanic acid (30 µg) (AMC) and ampicillin (10 µg) (AMP); (2) quinolones − ciprofloxacin (5 µg) (CIP); (3) phosphonic acids − fosfomycin (50 µg) (FOS); and (4) tetracyclines − tetracycline (30 µg) (TET). The plates were incubated for 24 h at 37 °C, and thereafter, the diameters of the inhibition zones formed were measured and classified as susceptible, resistant, or of intermediate sensitivity using the parameters adopted by the Clinical & Laboratory Standards Institute published in 2023 [11]. To be considered as a multi-drug-resistant (MDR) isolate, the criterion of being resistant to at least three antibiotic groups was used.

### 4.5. Statistical Analysis

Initially, the normality of each dataset was analyzed using the Shapiro–Wilk test utilizing the statistical software “R” version 4.2.3. Data that did not exhibit normal distribution (*p* < 0.05 in the Shapiro–Wilk test) were transformed into base 10 logarithms and subjected to the normality test again.

In cases where normal distribution, either without transformation or with logarithmic transformation, was observed, the counts of microorganisms from samples of different municipalities were compared through an analysis of variance. Subsequently, an analysis of the residuals of the linear model and a Shapiro–Wilk normality test were conducted. Additionally, the Breusch–Pagan test for variance heteroskedasticity was applied to verify whether the variance was constant (*p* > 0.05) utilizing the “car” package in “R” version 4.2.3. When the obtained linear model met these assumptions, multiple comparisons between the data from the municipalities were made using the Tukey test utilizing the “agricolae” package in “R” and adopting a confidence level of 95%. In cases where the data did not exhibit normal distribution or when the linear model did not meet the assumptions of the analysis of variance, a non-parametric analysis was employed through the Kruskal–Wallis rank sum test utilizing the “R” program version 4.2.3.

To perform comparisons between the counts of indicator microorganisms in the samples of shrimp and mussels, after checking the normality of the data using the Shapiro–Wilk test, the Wilcoxon rank sum exact test was used due to the absence of normality. Finally, for comparisons between samples of fresh or frozen mussels, after checking for normal distribution using the aforementioned test, the T-test was used when normal distribution was present, and the Wilcoxon rank sum exact test was used in the absence of normality.

## 5. Conclusions

It can be inferred that illegally marketed shrimp and mussels in the Vitória Region exhibit poor microbiological quality, attributed to them having elevated levels of mesophilic, psychrotrophic, and enterobacteria microorganisms. Although this issue is widespread throughout the study area, the shrimp samples showed higher microbial counts compared to the mussels, with fresh mussels also displaying higher counts than frozen ones. Furthermore, the potentially pathogenic bacterium *E. coli* was identified in these foods, alongside *E. coli* isolates a high frequency of resistance to β-lactams and one isolate that was simultaneously resistant to quinolones. This underscores the inherent risks to local public health associated with the consumption of these improperly prepared foods as sources of cross-contamination.

## Figures and Tables

**Table 1:** Mean counts (CFU/g) of mesophilic, psychrotrophic, and enterobacteria microorganisms in shrimp and mussels illegally sold without proper sanitary inspection in 2023 in the Vitória Region, Brazil.

Municipalities	Number of Samples	Mesophilic Microorganisms (CFU/g)	Psychrotrophic Microorganisms (CFU/g)	Enterobacteria (CFU/g)
	Shrimp samples			
Vitória	10	2.4 × 10^7^	3.9 × 10^8^	4.9 × 10^6^
Vila Velha	10	8.1 × 10^6^	1.5 × 10^9^	2.2 × 10^5^
Serra	10	1.3 × 10^7^	1.4 × 10^9^	1.2 × 10^6^
	Mussels samples			
Vitória	21	1.0 × 10^7^	8.3 × 10^7^	3.9 × 10^6^
Vila Velha	14	3.3 × 10^7^	1.5 × 10^8^	8.8 × 10^4^
Cariacica	4	2.7 × 10^6^	1.3 × 10^8^	4.4 × 10^4^

There is no legal limit for mesophilic, psychrotrophic, and enterobacteria microorganisms counts in shrimp and mussels sold to the population in Brazil.

**Table 2:** Results of sensitivity/resistance testing for 10 isolates of *E. coli* originating from shrimp (3) and mussel (7) samples illegally sold in Vitória Region, Brazil, concerning the antimicrobials amoxicillin + clavulanic acid (AMC), ampicillin (AMP), ciprofloxacin (CIP), fosfomycin (FOS), and tetracycline (TET). The isolates were classified as resistant (R), intermediate (I), and sensible (S) according to the CLSI’s 2023 guidelines [11].

Isolate	Sample	City	AMC	AMP	CIP	FOS	TET
1	Frozen Shrimp	Vitória	R	I	S	S	S
2	Frozen Shrimp	Vitória	I	R	S	S	S
3	Frozen Shrimp	Vila Velha	R	R	S	S	S
4	Fresh Mussel	Vila Velha	S	S	S	S	S
5	Fresh Mussel	Vitória	R	R	S	S	S
6	Fresh Mussel	Vitória	R	R	R	S	S
7	Frozen Mussel	Vila Velha	R	R	S	S	S
8	Frozen Mussel	Vitória	S	R	S	S	S
9	Frozen Mussel	Vila Velha	R	S	S	S	S
10	Frozen Mussel	Vitória	S	S	R	S	S

## Data Availability

Data will be available upon request.
